# The gut microbiota in liver transplantation recipients during the perioperative and postoperative recovery period

**DOI:** 10.3389/fmicb.2025.1684303

**Published:** 2025-11-28

**Authors:** Zhongyuan Bai, Yan Wang, Yaoping Li, Jun Xu, Zhiyong Lai

**Affiliations:** 1 Shanxi Medical University, Taiyuan, China; 2 First Clinical Medical School, Shanxi Medical University, Taiyuan, China; 3 Department of Colonrectal, Shanxi Provincial People's Hospital, Taiyuan, China; 4 Department of Hepatobiliary Surgery and Liver Transplantation Center, First Hospital of Shanxi Medical University, Taiyuan, China; 5 Shanxi Key Laboratory of Digestive Diseases and Organ Transplantation, First Hospital of Shanxi Medical University, Taiyuan, China; 6 Department of Biliopancreatic Surgery, First Hospital of Shanxi Medical University, Taiyuan, China

**Keywords:** Chronic Liver Disease, liver transplantation, gut microbiota, metagenomics, ABC transport

## Abstract

**Background:**

Chronic Liver Disease (CLD) is one of the frequent causes of death, especially in the developing world. Liver transplantation (LT) is an effective modality to treat end-stage liver disease. Perioperative management of liver transplantation patients and prevention of postoperative complications are the key to improving patient prognosis and quality of life, and the intestinal flora of these patients can affect postoperative complications and overall prognosis.

**Method:**

We collected a total of 151 fecal samples from 59 liver transplantation patients at different stages from the First Hospital of Shanxi Medical University. Using 16S rRNA sequencing technology, we compared the characteristics and changes of their microbiota. We selected 42 samples for metagenomic sequencing using the microPITA method to further analyze the composition and functional differences of the microbiota during the perioperative period of liver transplantation across various time points.

**Results:**

After liver transplantation (LT), the diversity of gut microbiota initially decreased and then increased. Firmicutes, Proteobacteria, and Bacteroidota were the main bacterial groups during the perioperative period. Firmicutes and Proteobacteria initially decreased and then increased, while Bacteroidota exhibited the opposite process. Alpha diversity and beta diversity analyses indicated that 1 month post-transplantation was a turning point for microbiota recovery (*P* < 0.01). Metagenomic sequencing, analyzed using the LEfSe method, identified a total of 50 genera that played significant roles in this process. The changes in microbiota exhibited the same trend as the 16S rRNA results. KEGG pathway analysis also indicated that 1 month was a critical time point, with Ko02010 potentially being a key pathway for recovery in LT patients, and it showed a negative correlation with Bacteroidota (*P* < 0.05).

**Conclusion:**

The diversity of intestinal flora in the perioperative period of LT patients decreased first and then increased, and the turning point of intestinal flora recovery was 1 month after LT surgery.

## Introduction

According to recent data, approximately 1.5 billion people worldwide were affected by Chronic Liver Disease (CLD) in 2024 (Burki, 2024). Each year, liver diseases account for approximately 2 million deaths globally, with liver cirrhosis being the 11th leading cause of mortality worldwide (Asrani et al., 2019). Hepatitis B virus (HBV) is a leading cause of mortality worldwide, contributing significantly to the development of cirrhosis, and hepatocellular carcinoma (HCC) (Nguyen et al., 2020). Additionally, the global prevalence of NAFLD is approximately 25%, making it a major contributor to both cirrhosis and HCC (Powell et al., 2021). Asia is one of the regions with the highest incidence of Chronic Liver Disease (CLD), and in China, the number of individuals affected by CLD may exceed 447 million. According to statistics, approximately 81.98 million people in China tested positive for HBsAg in 2022 (Polaris Observatory Collaborators, 2023).

Liver transplantation is an effective treatment for end-stage liver disease, with significant improvements in outcomes for patients with cirrhosis and hepatocellular carcinoma (HCC). Moreover, advancements in surgical techniques and the development of postoperative immunosuppressive therapies have greatly enhanced post-transplant recovery and survival rates. However, extending post-transplant survival and reducing perioperative and postoperative complications remain significant challenges in liver transplantation. Common postoperative complications include acute and chronic rejection, infections, bleeding, and biliary-related issues (Agostini et al., 2023). One of the primary challenges encountered by liver transplant recipients during the perioperative period is infection. Evidence indicates that colonization with multidrug-resistant bacteria (MDRB) can disrupt intestinal microbial homeostasis, and MDRB infection has been identified as a leading contributor to post-liver transplantation (LT) morbidity and mortality (Annavajhala et al., 2019). Recent studies suggest that fecal metabolite profiling may serve as a valuable tool for identifying LT patients at higher risk of postoperative infections and could provide actionable insights for microbiome-targeted therapeutic interventions (Lehmann et al., 2024). Postoperative recovery in liver transplant patients is often complicated by malnutrition, with reports indicating that more than half of liver transplant recipients experience nutritional deficiencies (Saiman and Serper, 2021). Additionally, long-term quality of life following liver transplantation is a commonly overlooked issue, with anxiety and depression emerging as major psychosocial complications (García-Alanís et al., 2021).

In recent years, the relationship between gut microbiota and liver physiology, metabolism, and pathological changes has been increasingly recognized and studied. The liver and gut are closely interconnected, as exemplified by the concept of the “liver-gut axis,” which highlights the strong connection between these two organs (Pabst et al., 2023). The development of CLD, as well as complications of cirrhosis such as hepatic encephalopathy, have also been confirmed to be associated with gut microbiota dysbiosis and disruption of the intestinal mucosal barrier (Hu et al., 2020; Won et al., 2022). The human gut microbiota is a vast ecosystem composed of bacteria, archaea, viruses, and fungi. These gut microorganisms play crucial roles in the human body, including modulating immune responses, facilitating digestion and metabolism, and regulating the proliferation and differentiation of epithelial cells. Furthermore, specific microbiota may influence the “brain-gut axis,” thereby affecting the patient's neurological function (Pabst et al., 2023; Zheng et al., 2019; Rothschild et al., 2018). Therefore, studying the gut microbiota in liver transplant patients during the perioperative period is of significant importance for the success of the transplantation, accelerating patient recovery, reducing postoperative complications, and improving the overall quality of life.

Currently, most studies on the gut microbiota in liver transplantation focus on basic experimental research, while some clinical evidence is limited to analyzing the differences in gut microbiota during the perioperative period in liver transplant patients (Banerjee et al., 2025). This study is the first to investigate the gut microbiota of liver transplant patients for more than 6 months post-surgery, using metagenomic sequencing, with the aim of identifying the intestinal metabolic characteristics at different time points following the transplantation.

We analyzed the gut microbiota of liver transplant patients during the perioperative and postoperative recovery periods using 16S rRNA sequencing and metagenomic sequencing technologies. And then, we compared the differences in gut microbiota between liver transplant patients at different time points and healthy controls and investigated the composition of the gut microbiota at various postoperative stages. We aim to characterize the overall distribution and differences in the gut microbiota during the perioperative and postoperative survival periods in liver transplant patients. The primary objective of this study is to identify the patterns of postoperative recovery in liver transplant patients, providing a basis for future rehabilitation and treatment strategies.

## Materials and methods

### Study population and sample collection

This study collected 120 fecal samples from 59 liver transplant patients during the perioperative period and postoperative period at the First Hospital of Shanxi Medical University between 2021 and 2023, as well as 31 samples from a control group. All 31 control group samples were selected from the patients' family members (such as spouses, brothers, etc.) as the sampling subjects. Exclusion criteria are as follows: (1) patients with concomitant inflammatory bowel disease, gastrointestinal tumors, or other organ diseases; (2) patients who had used antibiotics within 1 month prior to surgery and who were readmitted or used antibiotics again due to unstable conditions after surgery; and (3) patients who did not receive the same immunosuppressive regimen, which includes tacrolimus, sirolimus, mycophenolate mofetil, and methylprednisolone. All liver transplant patients who were treated with broad-spectrum antibiotics (such as cefoperazone sulbactam sodium, meropenem) during the operation and for 3 weeks after the operation were required to stop using them before discharge. Immunosuppressive therapy is a combined treatment approach of multiple immunosuppressants, mainly calcineurin inhibitors and mycophenolic acid inhibitors. We regularly follow up on the tacrolimus blood concentration of patients after the operation and adjust the target blood concentration range in a timely manner. It is 8–12 ng/mL in the first 3 months after transplantation and 7–10 ng/mL in 3–6 months. It is 6–8 ng/mL for 6–12 months. Following standardized protocols, all stool samples were put into sterile containers to immediately snap-frozee in liquid nitrogen within 4 h of collection and subsequently refrigerated at −80 °C. The study design and procedures complied with the Declaration of Helsinki, and the research protocol was approved by our hospital's ethics committee. We grouped the 151 samples as follows: before-liver transplantation (BLT: 20); 1 week post-liver transplantation (LT1W: 20); 2 week post-liver transplantation (LT2W: 20); 1 month post-liver transplantation (LT1M: 20); 3 month post-liver transplantation (LT3M: 20); 6 month post-liver transplantation (LT6M: 20); and healthy controls (GC: 31).

### Sampling and DNA extraction

To isolate genomic DNA, we processed fecal samples using a fecal genomic DNA extraction kit (TianGen, China, Catalog #: DP712). The purity and integrity of the extracted DNA were then assessed by 1% agarose gel electrophoresis. Appropriate sample DNA was placed in a centrifuge tube and diluted to 1 ng/μL with sterile water.

### 16S rRNA gene amplicon sequencing

We prerformed 16S rRNA sequencing using the isolated fecal DNA as a template for amplification. The bacterial primers 515F and 806R were used to amplify the V4 region of the 16S rRNA gene via PCR. All PCR reactions were carried out with 15 μL of Phusion^®^ High Fidelity PCR Master Mix (New England Biolabs); 0.2 μM of forward and reverse primers, and about 10 ng template DNA. The reaction was conducted under the following conditions: initial denaturation at 98 °C for 60 s, followed by 35 cycles of denaturation at 98 °C for 10 s, annealing at 50 °C for 30 s, and extension at 72 °C for 30 s, with a final extension at 72 °C for 5 min. The PCR products were analyzed by electrophoresis on a 2% agarose gel. The qualifying PCR products were then purified using magnetic beads and quantified by enzyme-linked immunosorbent assay (ELISA). The concentration of the PCR products was determined. Then, mixture PCR products were purified with Universal DNA Purification Kit (TianGen, China, Catalog #: DP214). Using the NEB Next^®^ Ultra™ II FS DNA PCR-free Library Prep Kit (New England Biolabs) conducted library construction, and the constructed library was quantified by Qubit and Q-PCR. The PE 250 was sequenced using NovaSeq 6000.

### Bioinformatics analysis and statistical processing

According to the Barcode sequence and PCR amplification primer sequence, the sample data were separated from the disembarkation data. After the Barcode and primer sequences were cut off, the reads of each sample were spliced using FLASH, and the spliced sequences were Raw Tags data. The fastp software (Version 0.23.1) is used to process the Raw Tags obtained by splicing through strict filtering to obtain high-quality Tags data (Clean Tags). After the above processing, the resulting tags were subjected to chimeric sequence removal. The tag sequences were compared against a species annotation database (silva database https://www.arb-silva.de/for 16S/18S, Unite database https://unite.ut.ee/ for ITS) to identify chimeric sequences, which were then gotten the final Effective Tags.

The effective tags obtained were denoised using the DADA2 module or Deblur (DADA2 is used by default) in QIIME2 (Version Qiime2-202006) software to generate the final ASVs (Amplicon Sequence Variants) and feature table. Species annotation, phylogenetic tree construction, data normalization, and species abundance statistics were performed using QIIME2 software. Venn Diagram and Flower plot were generated using the VennDiagram function in R and the SVG function in Perl, respectively. Phylogenetic trees in SVG format were plotted using Perl. Observed Features, Shannon, and Chao1 index were calculated using QIIME2 software. Box plots and rarefaction curves were then generated using R package. Beta diversity analysis was performed in QIIME2 based on both weighted and unweighted distances. PCA and PCoA analyses were conducted using the ade4 package and ggplot2 package in R software (version 4.0.3).

### Metagenomic sequencing and quality control

We selected Metagenomic sequencing samples using the Discriminant method in microPITA (Supplementary Figure S1). We chose six samples from the central position of each group, for a total of 42 samples, and then performed metagenomic sequencing. The samples selected from the BLT group were designated as Group A, samples from the LT1W group as Group B, samples from the LT2W group as Group C, and samples from the LT1M group as Group D, The samples from LT3M group as Group E; The samples from group LT6M as Group S; The samples from group GC to Group G.

DNA samples were tested by agarose gel electrophoresis (AGE) to analyze the purity and integrity of DNA. For qualified samples, 1 μg of genomic DNA was randomly broken into fragments of about 350 bp in length using a Covaris ultrasonic disruptor for library construction. The entire library preparation was completed through end repair, A-tailing, sequencing adapter addition, purification, PCR amplification, and other steps. After the library was constructed, Qubit2.0 was used for preliminary quantification, and the library was diluted to 2 ng/ul. Then, the insert size of the library was detected using Agilent 2100. After the insert size met expectations, the effective concentration of the library was accurately quantified using the Q-PCR method (library effective concentration>3 nM) to ensure the quality of the library. After the library was qualified, different libraries were pooled according to the effective concentration and the target data volume requirements for Illumina PE150 sequencing.

### Metagenomic bioinformatics analysis

#### Preprocessing of sequencing results and assembly of metagenome

Readfq (https://github.com/cjfields/readfq) is used to preprocess Raw Data from the Illumina sequencing platform to obtain Clean Data for subsequent analysis. The specific steps are as follows: (1) remove reads with low-quality bases (default quality threshold is < =38) that exceed a certain proportion (default length is 40 bp); (2) remove reads with N bases reaching a certain proportion (default length is 10 bp); (3) remove reads whose overlaps with adapters exceed a certain threshold (default length is 15 bp). MEGAHIT software was used to assemble and analyze the Clean Data, and then the assembled Scaffolds were interrupted at the N junction to obtain Scaftigs without N. Next, for Scaftigs generated from a single-sample assembly, we filtered out fragments shorter than 500 bp and then performed statistical analysis and subsequent gene analysis.

### Gene prediction and abundance analysis

With the default parameters, MetaGeneMark (http://topaz.gatech.edu/GeneMark/) is used to perform ORF prediction for Scaftigs (≥500 bp) of each sample and the information with a length less than 100 nt in the prediction results is filtered out. For the ORF prediction results, CD-HIT software (http://www.bioinformatics.org/cd-hit/) is used to eliminate redundancy and obtain the non redundant initial gene catalog. Clean Data of each sample is aligned to the initial gene catalog by using Bowtie2 to calculate the number of reads of the genes on each sample alignment. Genes with reads ≤ 2 in each sample are filtered out to finally determine the gene catalog (Unigenes) for subsequent analysis. Based on the number of reads aligned and the length of the gene, the abundance of each gene in each sample is calculated. Based on the abundance of each gene in the gene catalog in each sample, basic information statistics, correlation analysis between samples and groups, and Venn diagram analysis of gene number are performed.

### Species annotation

We used DIAMOND software (https://github.com/bbuchfink/diamond/) for the sequence alignment of Unigenes with those of bacteria, fungi, archaea, and viruses extracted from NCBI's NR database (https://www.ncbi.nlm.nih.gov/).

Based on the abundance table at each classification level, Krona analysis was carried out, and the relative abundance overview was displayed. PCA (R ade4 package) and NMDS (R vegan package) were analyzed for dimensionality reduction. Anosim analysis (R vegan package) was used to check the differences between groups. LEfSe analysis was then used to search for species differences between groups. LEfSe analysis was performed using LEfSe software (LDA Score is four by default).

### Annotations of common functional database

We used DIAMOND software (https://github.com/bbuchfink/diamond/) to compare the Unigenes and KEGG database function. For the comparison results of each sequence, the comparison results with the highest score were selected for subsequent analysis. Based on the comparison results, the relative abundance of different functional levels was calculated (the relative abundance of each functional level was equal to the sum of the relative abundance of genes annotated as the functional level). Based on the abundance table at each classification level, the number of annotated genes were counted, relative abundance overview display, abundance cluster heat map display, PCA and NMDS dimension reduction analysis, Anosim inter-group (within) difference analysis based on functional abundance, and Metastat analysis of inter-group functional difference were performed.

### Statistical analysis

Statistical methods: We performed statistical analyses using R 4.3.0 language and SPSS version 26.0. Spearman's rank correlation test was used for correlation analysis. Differences in species and functional profiles between groups were evaluated using the Kruskal-Wallis rank-sum test. FDR correction was performed for all comparisons among multiple groups. *P* < 0.05 was considered statistically significant.

## Result

**Figure 1:**
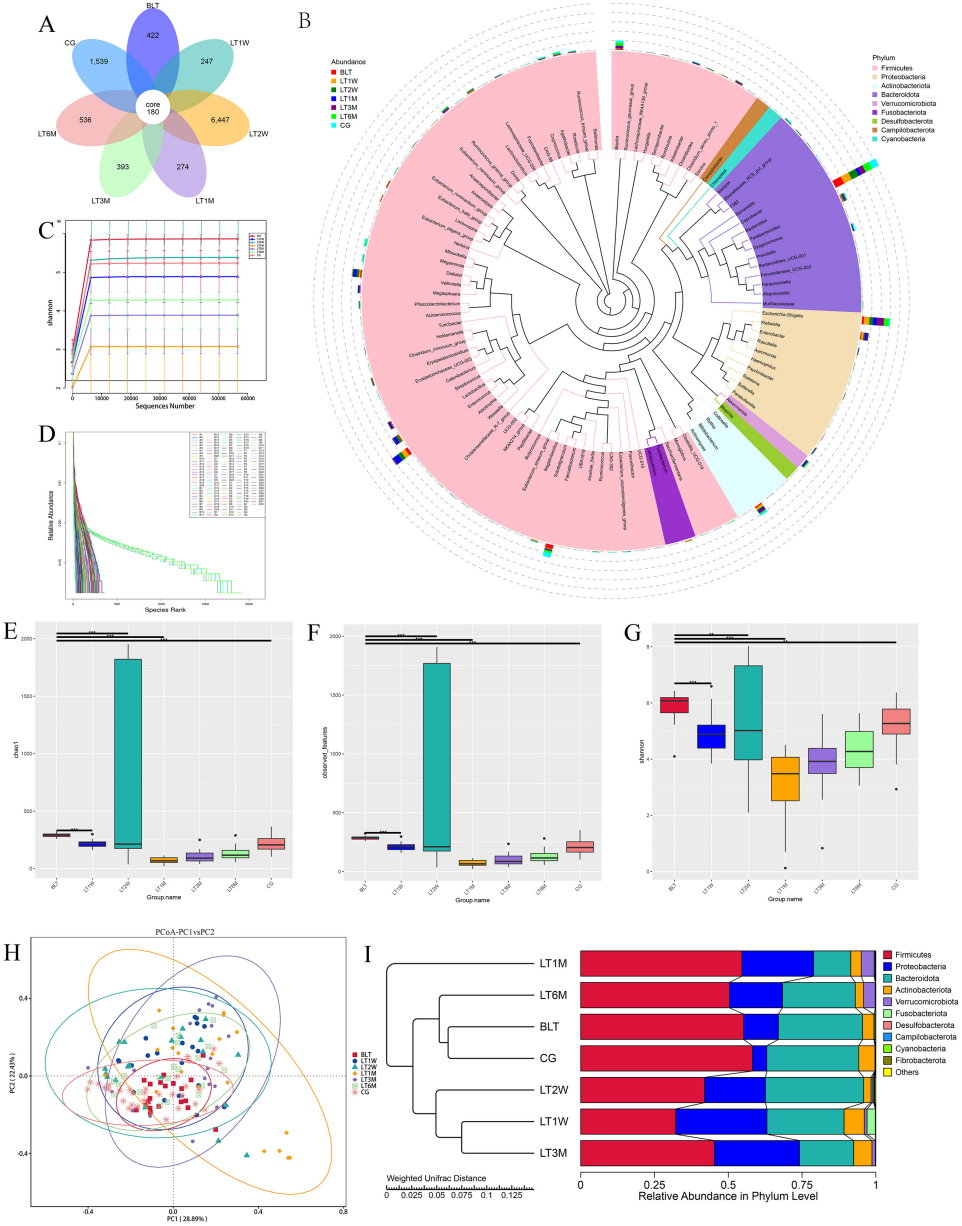
Results of 16S rRNA sequencing, α diversity and β diversity results. **(A)** Venn diagram; **(B)** Genus level species evolutionary tree; **(C)** Sample dilution curve of each group during perioperative period of liver transplantation; **(D)** Sample abundance curve of each sample during perioperative liver transplantation; **(E)** chao1 index; **(F)** Observation feature index; **(G)** Shannon index (* indicates *p* < 0.05; ** means *p* < 0.01; *** means *p* < 0.001); **(H)** The diversity analysis results showed; **(I)** UPGMA cluster analysis diagram based on PCoA analysis results.

In this study, we collected 151 liver transplant (LT) patient samples. The baseline characteristics of the participants for each sample are presented in Table 1. It can be observed that the clinical parameters (Hb, WBC, PLT, TB, DB, IB, TBA, PT, D-Dimer, and PA) of liver transplant (LT) patients show significant changes (*p* < 0.05) during the perioperative and postoperative recovery periods. These indicators suggest that liver transplant patients experienced significant improvements in both hematological parameters and liver function after transplantation. In addition, among the 31 patients in the GC group, there were male (58.06%) and female (41.93%), with an average age of 51.55±10.15. The above content has been modified in the text. Then our study employed 16S rRNA sequencing to analyze 151 intestinal microbiota samples, aiming to identify the patterns of gut microbiota changes during the perioperative and postoperative recovery periods in liver transplant (LT) patients. V3-V4 hypervariable regions of the samples were sequenced using the NovaSeq sequencing platform. After stringent quality control of the sequencing results, we performed denoising using QIIME2 software, generating the final identification of 10,038 ASVs (Amplicon Sequence Variants). After constructing the Venn diagram (Figure 1A), we observed that 180 ASVs were shared among the seven groups, with 422 ASVs unique to the BLT group, 247 ASVs unique to the LT1W group; 6,447 ASVs unique to LT2W group; 274 ASVs unique to LT1M group; 393 ASVs unique to LT3M group; 256 274 ASVs unique to LT6M group; and 1,539 ASVs unique to GC group. Subsequently, species annotation was performed on all ASVs, and a phylogenetic tree at the genus level of the top 100 species was constructed (Figure 1B). From these results, we can infer that the gut microbiota in each group are closely related in origin, with the majority originating from the Firmicutes phylum (the old NCBI hierarchical classification). However, in the postoperative LT1W, LT2W, and LT1M groups, besides firmicutes, bacteroidota and Proteobacteria also originated. The rarefaction curve (Figure 1C) reflects the sequencing depth. The curve leveling off indicates that the sequencing depth is sufficient and the data volume is adequate. Based on each sample's total number of ASV sequences, we ranked the ASVs by abundance from high to low and generated a species abundance curve (Figure 1D). On the x-axis, the distribution of the LT2W group appears wider, with a more gradual decline, which suggests that this group has higher species abundance and greater evenness. The LT1M group exhibited the narrowest distribution and the steepest decline, indicating a lower species richness and reduced evenness in microbial abundance. Additionally, the species abundance in the LT6M group was closest to that in the GC group.

**Table 1:** Subject baseline characteristics.

**Clinical characteristics**	**BLT (*n* = 20) (%)**	**LT1W (*n* = 20) (%)**	**LT2W (*n* = 20) (%)**	**LT1M (*n* = 20) (%)**	**LT3M (*n* = 20) (%)**	**LT6M (*n* = 20) (%)**	***p***
Gender							0.279
Male	14 (70)	12 (60)	11 (55)	17 (85)	13 (65)	16 (80)	
Female	6 (30)	8 (40)	9 (45)	3 (15)	7 (35)	4 (20)	
Age, yrs	52.80 ± 10.30	54.00 ± 7.33	54.35 ± 7.67	52.3 ± 11.09	52.65 ± 10.18	54.20 ± 10.04	0.971
BMI	23.13 ± 4.27	22.49 ± 3.68	22.37 ± 3.36	24.07 ± 4.27	24.45 ± 3.76	23.72 ± 4.17	0.463
Diagnose							0.921
NAFLD	13 (65)	14 (70)	15 (75)	12 (60)	14 (70)	14 (70)	
AFLD	5 (25)	2 (10)	3 (15)	5 (25)	2 (10)	3 (15)	
Cancer	2 (10)	4 (20)	2 (10)	3 (15)	4 (20)	3 (15)	
Etiology							0.981
HBV	11 (55)	12 (60)	10 (50)	13 (65)	14 (70)	12 (60)	
Alcoholic cirrhosis	5 (25)	2 (10)	3 (15)	5 (25)	2 (10)	3 (15)	
Drug-induced liver cirrhosis	1 (5)	1 (5)	0 (0)	0 (0)	0 (0)	1 (5)	
Immune cirrhosis	1 (5)	5 (25)	6 (30)	1 (5)	3 (15)	3 (15)	
Biliary cirrhosis	2 (10)	0 (0)	1 (5)	1 (5)	1 (5)	1 (5)	
Ascittes							0.745
+	13 (65)	11 (55)	10 (50)	10 (50)	8 (40)	11 (55)	
–	7 (35)	9 (45)	10 (50)	10 (50)	12 (60)	9 (45)	
Splenauxe							0.979
+	14 (70)	15 (75)	15 (75)	16 (80)	15 (75)	16 (80)	
-	6 (30)	5 (25)	5 (25)	4 (20)	5 (25)	4 (20)	
RBC, ^*^10^12/*L*^	3.61 ± 0.82	3.77 ± 0.95	3.27 ± 0.88	3.80 ± 0.87	4.30 ± 0.65	13.57 ± 39.65	0.299
Hb, g/L	108.55 ± 22.52	116.95 ± 22.68	101.80 ± 19.84	117.50 ± 24.63	129.70 ± 19.43	142.00 ± 20.06	0.000^*^
WBC, ^*^10^9/*L*^	4.39 ± 3.66	6.875 ± 3.90	5.95 ± 2.29	4.43 ± 1.38	4.39 ± 2.13	4.75 ± 1.95	0.015^*^
PLT, ^*^10^9/*L*^	52.50 (37.00–73.50)	76.50 (39.00–117.75)	147.00 (105.50–170.00)	121.00 (85.75–170.50)	99.50 (80.00–178.75)	132.00 (92.75–180.75)	0.000^*^
TB, μmol/L	92.49 ± 145.11	68.96 ± 58.72	49.95 ± 33.28	31.79 ± 26.94	15.52 ± 8.73	14.01 ± 3.80	0.001^*^
DB, μmol/L	46.80 ± 94.76	33.82 ± 32.31	25.13 ± 20.60	13.21 ± 13.96	4.46 ± 3.13	3.99 ± 1.42	0.008^*^
IB, μmol/L	44.23 ± 51.70	35.14 ± 26.80	24.82 ± 13.52	18.84 ± 14.04	11.06 ± 6.17	10.03 ± 2.86	0.000^*^
ALT, U/L	27.50 (17.25–107.75)	85.00 (44.25–131.25	61.50 (25.75–176.00)	36.00 (19.50–83.50)	12.50 (9.25–23.00)	18.00 (9.75–21.75)	0.101
AST, U/L	43.00 (25.00–115.75)	25.50 (20.00–51.50	31.00 (23.25–75.50)	22.50 (17.00–35.75)	16.50 (14.00–21.75)	19.00 (15.25–23.75)	0.159
TBA, μmol/L	111.21 ± 137.75	5.83 ± 4.33	7.35 ± 9.58	15.28 ± 8.32	15.71 ± 6.10	12.5 ± 67.78	0.000^*^
PT, s	20.37 ± 4.81	15.92 ± 2.62	14.74 ± 1.71	18.57 ± 2.46	15.08 ± 2.27	14.06 ± 2.31	0.000^*^
DDimer, mg/L	91.50 (3.265–748.00)	1078.50 (7.57–1708.25)	984.50 (690.00–161.25)	517.50 (144.25–1036.75)	63.50 (9.25–95.00)	81.50 (52.50–119.00)	0.042^*^
ALB, g/L	32.75 ± 5.53	38.55 ± 11.23	40.02 ± 5.52	42.84 ± 6.17	41.83 ± 6.61	45.16 ± 9.42	0.920
PA, mg/L	104 ± 44.22	238.27 ± 103.66	250.20 ± 95.70	254.40 ± 83.55	247.40 ± 59.23	246.09 ± 84.53	0.000^*^

All results were corrected by FDR. ^*^ means *p* < 0.05.

## Diversity analysis

### Alpha diversity analysis

Alpha diversity was analyzed by using Chao1, Observed features, and shannon diversity index, which, respectively, represent the richness, evenness, and total number of observed species in each group (Figures 1E–G). All three observed indices displayed a “V” shaped pattern. The Chao1, Observed features, and Shannon diversity index were highest in the CG and BLT groups, while the LT1M group exhibited the lowest values. Based on the Kruskal-Wallis (K-W) test (Table 2), we observed significant differences (*p* < 0.05) between the BLT group and the LT1M group in all three observed indices compared to the other groups. Significant differences (*p* < 0.05) were observed between the CG group and the LT1M, LT3M, and LT6M groups. However, no significant differences were found between the LT1W and LT2W groups, as well as between the LT3M and LT6M groups. The analysis of alpha diversity effectively reflects the changes in gut microbiota characteristics during the perioperative and postoperative periods in liver transplant patients. Liver transplantation has a significant impact on the diversity and richness of the gut microbiota. Therefore, differences were observed between the BLT group and all other groups. The difference between the BLT and CG groups may be related to liver disease. From 1 week to 1 month after the surgery, a significant decline in gut microbiota diversity and abundance was observed (no significant differences were found between the LT1W and LT2W groups). One month after surgery, these two values reached at their lowest, which may be related to the surgical impact on the patients and the use of antibiotics during their hospital stay. As patients gradually recover, the use of antibiotics and immunosuppressants decreases, leading to a gradual restoration of gut microbiota diversity and abundance (No significant differences were found between the LT3M and LT6M groups). Significant differences remained between the LT6M group and the GC group, which may be related to the long-term use of immunosuppressants. This also suggests that 6 months after the transplantation may not represent the endpoint of gut microbiota recovery in these patients (the composition of key microbiota and alpha diversity). It requires further long-term studies to determine whether the patients' gut microbiota can recover to levels comparable to the CG group. Therefore, for patients with end-stage liver disease, the most significant impact of liver transplantation on the gut microbiota occurs during the period from 1 week to 1 month after the surgery. Moreover, 1 month is the time point at which gut microbiota abundance is at its lowest. Identifying critical time points and maintaining gut microbiota stability during these periods is essential, as it significantly contributes to enhancing postoperative recovery and minimizing complications in liver transplant patients.

**Table 2:** Chao1 observed_features shannon index analyzed with Kruskal–Wallis rank sum.

	**Chao1**			**Observed_features**			**Shannon**		
**Comparison**	**Difference**	***p*** **-value**	**Signif**.	**Difference**	***p*** **-value**	**Signif**.	**Difference**	***p*** **-value**	**Signif**.
BLT - CG	36.29	0.00	^***^	37.52	0.00	^***^	25.19	0.01	^**^
BLT - LT1M	106.58	0.00	^***^	106.93	0.00	^***^	95.55	0.00	^***^
BLT - LT1W	35.40	0.00	^***^	35.78	0.00	^***^	41.95	0.00	^***^
BLT - LT2W	34.38	0.00	^***^	35.15	0.00	^***^	34.15	0.00	^**^
BLT - LT3M	88.73	0.00	^***^	89.78	0.00	^***^	75.88	0.00	^***^
BLT - LT6M	75.43	0.00	^***^	75.88	0.00	^***^	64.13	0.00	^***^
CG - LT1M	70.28	0.00	^***^	69.40	0.00	^***^	70.36	0.00	^***^
CG - LT1W	−0.89	0.91		−1.75	0.83		16.76	0.08	.
CG - LT2W	−1.92	0.81		−2.37	0.77		8.96	0.34	
CG - LT3M	52.43	0.00	^***^	52.25	0.00	^***^	50.68	0.00	^***^
CG - LT6M	39.13	0.00	^***^	38.35	0.00	^***^	38.93	0.00	^***^
LT1M - LT1W	−71.18	0.00	^***^	−71.15	0.00	^***^	−53.60	0.00	^***^
LT1M - LT2W	−72.20	0.00	^***^	−71.78	0.00	^***^	−61.40	0.00	^***^
LT1M - LT3M	−17.85	0.05	^*^	−17.15	0.06	.	−19.68	0.06	.
LT1M - LT6M	−31.15	0.00	^***^	−31.05	0.00	^***^	−31.43	0.00	^**^
LT1W - LT2W	−1.02	0.91		−0.63	0.94		−7.80	0.45	
LT1W - LT3M	53.33	0.00	^***^	54.00	0.00	^***^	33.93	0.00	^**^
LT1W - LT6M	40.03	0.00	^***^	40.10	0.00	^***^	22.18	0.03	^*^
LT2W - LT3M	54.35	0.00	^***^	54.63	0.00	^***^	41.73	0.00	^***^
LT2W - LT6M	41.05	0.00	^***^	40.73	0.00	^***^	29.98	0.00	^**^
LT3M - LT6M	−13.30	0.14		−13.90	0.12		−11.75	0.26	

^*^ means *p* < 0.05; ^**^ means *p* < 0.01; ^***^ means *p* < 0.001.

### Beta diversity analysis

Beta diversity (the similarity in community composition between species) was assessed using PCoA. According to the PCoA results (Figure 1H), PCoA analysis was performed based on Weighted UniFrac distances. If the samples are closer in distance, it means that the species composition is more similar. Therefore, samples with high community structure similarity tend to cluster together, while samples with significant community differences are located farther apart. We observed that after liver transplantation, the dispersion of samples gradually increased, reflecting an increase in beta diversity. Beta diversity in patients gradually increased, reaching its peak at 1 month post-surgery. Subsequently, the Beta diversity of patients gradually converged toward that of the GC group. Similar to Alpha diversity result, after liver transplantation, the gut microbiota diversity in the LT group began to increase and peaked around 1 month post-surgery, then gradually returned to levels comparable to the GC group.

To investigate the similarity between different groups, we performed UPGMA clustering analysis using the Weighted Unifrac distance matrix. The clustering results were integrated with the relative abundance of species at the phylum level for each sample, as shown in Figure 1I. The clustering results show that the BLT group is most closely associated with the GC group, followed by the LT6M group, while the LT1M group has the lowest degree of association. This indicates that the period when the LT patients' gut microbiota is closest to that of the control group is 6 months after liver transplantation, and that 1 month post-surgery is the time point when the gut microbiota in LT patients is most unstable and susceptible to disturbance.

### Species composition

Based on species annotation results at each taxonomic level, we selected the top 10 species with the highest relative abundances at the phylum, class, order, family, and genus levels for each group (Table 3) to plot bar charts that display the relative abundance of species annotation results at different taxonomic levels for each group.

**Table 3:** Through 16s rRNA sequencing, the species abundance of patients in perioperative liver transplantation groups was displayed at the level of kingdom, phylum, class, order, family and genus.

**Taxonomy**	**BLT**	**LT1W**	**LT2W**	**LT1M**	**LT3M**	**LT6M**	**CG**
**Phylum level**							
Firmicutes	55.20%	32.21%	41.98%	54.62%	45.28%	50.37%	58.16%
Proteobacteria	11.78%	30.90%	20.64%	24.33%	28.89%	18.01%	4.89%
Bacteroidota	28.53%	26.14%	33.27%	12.56%	18.34%	24.67%	31.15%
Actinobacteriota	3.82%	6.85%	2.48%	3.56%	6.20%	2.81%	5.50%
Verrucomicrobiota	0.18%	0.94%	0.47%	4.37%	1.14%	3.93%	0.12%
Fusobacteriota	0.39%	2.82%	0.42%	0.29%	0.01%	0.15%	0.08%
Desulfobacterota	0.05%	0.03%	0.06%	0.15%	0.09%	0.04%	0.08%
Campilobacterota	0.04%	0.11%	0.05%	0.01%	0.00%	0.00%	0.00%
Cyanobacteria	0.00%	0.01%	0.07%	0.08%	0.00%	0.00%	0.00%
Fibrobacterota	0.00%	0.00%	0.22%	0.00%	0.00%	0.00%	0.00%
Others	0.00%	0.00%	0.34%	0.03%	0.05%	0.02%	0.01%
**Class level**							
Bacilli	14.29%	15.19%	20.62%	30.41%	12.93%	3.07%	2.52%
Gammaproteobacteria	11.78%	30.90%	20.56%	24.32%	28.88%	18.01%	4.88%
Clostridia	34.41%	12.45%	12.44%	10.62%	28.49%	39.91%	45.49%
Bacteroidia	28.53%	26.14%	33.27%	12.56%	18.34%	24.67%	31.15%
Actinobacteria	3.78%	6.84%	2.45%	3.46%	6.14%	2.65%	5.16%
Negativicutes	6.51%	4.57%	8.90%	13.59%	3.86%	7.39%	10.15%
Verrucomicrobiae	0.18%	0.94%	0.43%	4.37%	1.14%	3.93%	0.12%
Fusobacteriia	0.39%	2.82%	0.42%	0.29%	0.01%	0.15%	0.08%
Desulfovibrionia	0.05%	0.03%	0.04%	0.15%	0.09%	0.04%	0.08%
Campylobacteria	0.04%	0.11%	0.05%	0.01%	0.00%	0.00%	0.00%
Others	0.04%	0.03%	0.79%	0.22%	0.12%	0.17%	0.36%
**Order level**							
Lactobacillales	13.51%	14.62%	17.90%	29.51%	10.48%	1.66%	1.37%
Enterobacterales	10.04%	29.92%	18.99%	22.25%	28.07%	16.75%	3.91%
Bacteroidales	28.53%	26.14%	33.26%	12.56%	18.34%	24.67%	31.15%
Bifidobacteriales	3.75%	6.79%	2.41%	2.95%	6.00%	2.63%	5.15%
Veillonellales-Selenomonadales	5.98%	4.15%	7.95%	12.81%	3.25%	6.21%	8.80%
Verrucomicrobiales	0.18%	0.94%	0.43%	4.37%	1.14%	3.93%	0.12%
Lachnospirales	19.48%	7.46%	4.97%	6.92%	15.97%	24.84%	24.20%
Oscillospirales	12.78%	3.24%	4.47%	1.79%	5.13%	10.18%	18.45%
Burkholderiales	1.10%	0.70%	1.05%	1.91%	0.38%	0.63%	0.61%
Erysipelotrichales	0.74%	0.55%	2.55%	0.77%	2.43%	1.39%	1.07%
Others	3.92%	5.48%	6.03%	4.15%	8.80%	7.11%	5.16%
**Family level**							
Enterococcaceae	8.52%	12.83%	10.73%	17.12%	3.86%	0.68%	0.25%
Enterobacteriaceae	10.01%	29.89%	18.91%	22.23%	28.04%	16.67%	3.90%
Lactobacillaceae	2.63%	1.08%	6.50%	7.88%	3.73%	0.34%	0.76%
Bifidobacteriaceae	3.75%	6.79%	2.41%	2.95%	6.00%	2.63%	5.15%
Bacteroidaceae	26.38%	23.74%	21.69%	7.98%	14.29%	19.80%	21.09%
Selenomonadaceae	0.00%	0.00%	0.08%	0.00%	0.12%	2.89%	5.14%
Akkermansiaceae	0.18%	0.94%	0.43%	4.37%	1.14%	3.93%	0.12%
Prevotellaceae	0.28%	0.06%	2.71%	1.50%	0.81%	0.19%	4.90%
Lachnospiraceae	19.48%	7.46%	4.96%	6.92%	15.97%	24.83%	24.20%
Veillonellaceae	5.97%	4.15%	7.87%	12.81%	3.12%	3.29%	3.67%
Others	22.80%	13.05%	23.73%	16.24%	22.91%	24.74%	30.83%
**Genus level**							
Enterococcus	8.52%	12.83%	10.73%	17.12%	3.86%	0.68%	0.25%
Escherichia-Shigella	5.50%	14.98%	9.70%	11.16%	19.75%	14.03%	3.52%
Lactobacillus	2.63%	1.07%	6.46%	7.87%	3.73%	0.34%	0.76%
Bifidobacterium	3.73%	6.79%	2.41%	2.95%	6.00%	2.63%	5.15%
Klebsiella	2.78%	5.92%	3.69%	5.64%	4.33%	0.65%	0.11%
Bacteroides	26.38%	23.74%	21.69%	7.98%	14.29%	19.80%	21.09%
Megamonas	0.00%	0.00%	0.00%	0.00%	0.00%	2.85%	5.13%
Akkermansia	0.18%	0.94%	0.43%	4.37%	1.14%	3.93%	0.12%
Prevotella	0.28%	0.06%	1.38%	1.50%	0.80%	0.00%	4.53%
Megasphaera	1.63%	0.34%	0.86%	3.96%	0.58%	0.39%	0.43%
Others	48.37%	33.34%	42.66%	37.46%	45.52%	54.69%	58.91%

#### Phylum level

**Figure 2:**
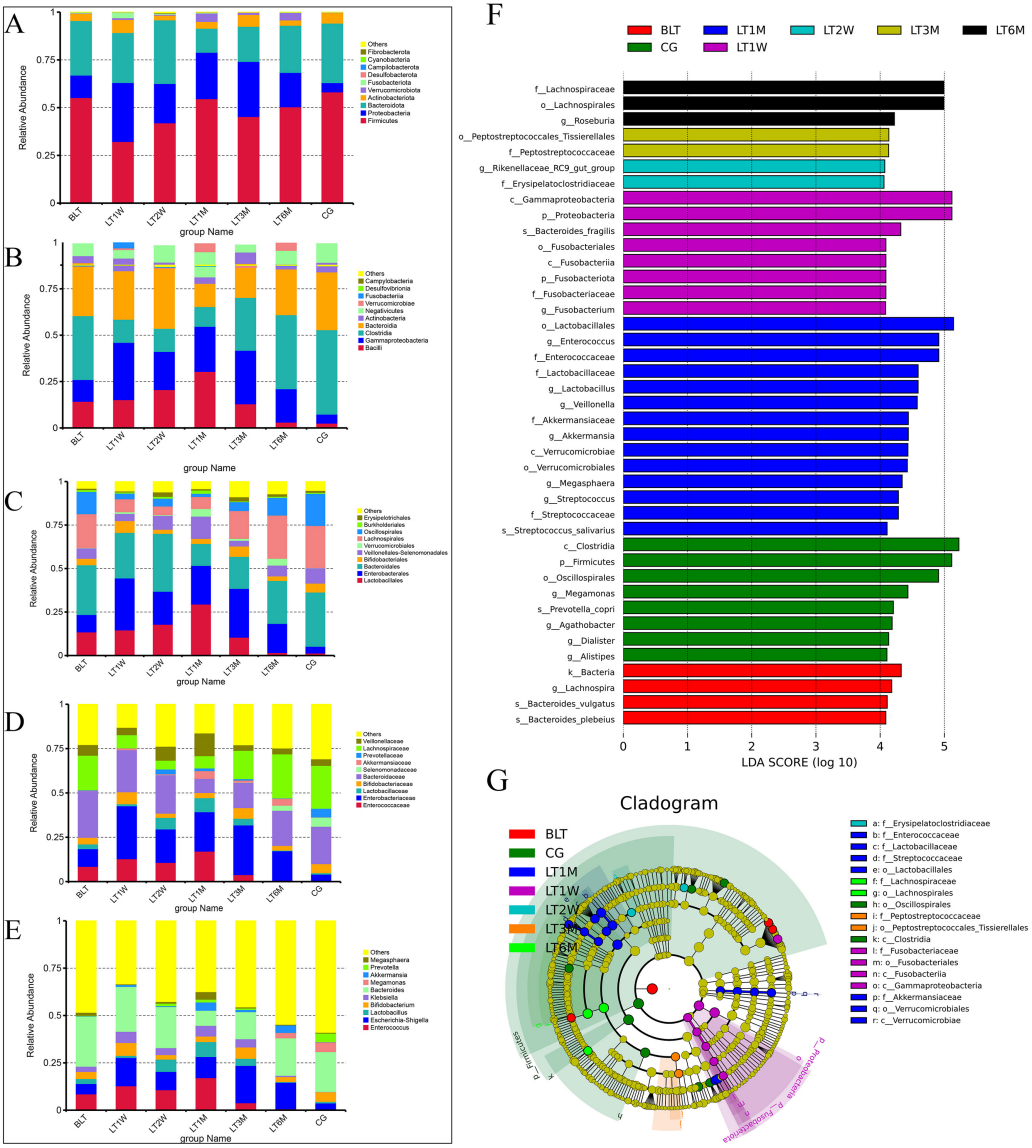
Species abundance cluster and LEfSe plot of all levels in the perioperative period of liver transplantation by 16S rRNA sequencing. **(A)** Phylum level species abundance between groups during perioperative period of liver transplantation; **(B)** Class level species abundance between groups during perioperative period of liver transplantation; **(C)** Order level species abundance between groups during perioperative period of liver transplantation; **(D)** Family level species abundance between groups during perioperative period of liver transplantation; **(E)** Genus level species abundance between groups during perioperative period of liver transplantation; **(F)** LEfSe analysis of bar chart; **(G)** LEfse analysis of evolutionary cladistics.

At the phylum level (Figure 2A), Firmicutes, Proteobacteria, and Bacteroidota were dominant across all groups. After liver transplantation, Firmicutes showed a significant decline initially (BLT: 55.20%; LT1W: 32.21%), followed by an increasing trend, and stabilized around 1 month post-surgery (LT1M:54.62%). Although Proteobacteria showed a decreasing trend post-surgery, it remained at relatively higher levels compared to the GC group. Bacteroidota exhibited a relatively low proportion one month post-surgery, with no significant differences in relative abundance compared to the GC group at other time points.

#### Class level

At the class level (Figure 2B), we further refined the classification of the gut microbiota, with the dominant groups being Bacilli, Gammaproteobacteria, Clostridia, and Bacteroidia. Bacilli peaked at 1 month after surgery, and by 6 months post-surgery, its abundance nearly returned to levels comparable to those in the GC group (LT1M: 30.41%; LT6M: 3.07%; GC: 2.52%).Clostridia showed that the proportion of the microbiota first decreased after the operation, but began to increase 1 month after the operation (4.88% vs. 25.33%), and then recovered to baseline levels around 6 months post-surgery. Bacteroidia followed a similar trend to Bacteroidota at the phylum level. Proteobacteria remained at relatively high levels throughout the 6-month postoperative period.

#### Order level

At the order level (Figure 2C), the most predominant bacterial orders were Bacteroidales, Enterobacterales, Lactobacillales, and Lachnospirales. The trends in the changes of Bacteroidales, Enterobacterales, and Lactobacillales are consistent with those of their respective higher taxonomic levels. It is noteworthy that Lachnospirales constituted a relatively low proportion from pre-surgery to 3 months post-surgery, reaching its lowest level at 1 month post-surgery, and subsequently recovering to levels comparable to those in the GC group by 6 months post-surgery (LT1M: 6.92%; LT6M: 2.48%; GC: 2.42%).

#### Family level

At the family level (Figure 2D), the gut microbiota was further characterized, with the dominant families being Enterococcaceae, Enterobacteriaceae, Bacteroidaceae, and Selenomonadaceae. Enterococcaceae, a family associated with infection, peaked 1 month post-surgery, and by 6 months post-surgery, its abundance had nearly returned to baseline levels, showing no significant difference compared to healthy controls (LT1M: 17.11%; LT6M: 0.68%; GC: 0.25%). As for the beneficial microbiota, Lachnospiraceae, which is involved in the metabolism of various carbohydrates, decreased from the postoperative period to 1 month post-surgery, then gradually recovered to baseline levels around 6 months post-surgery.

#### Genus level

At the genus level (Figure 2E), the dominant genera were Bacteroides and Enterococcus. Of particular interest, the opportunistic pathogen Klebsiella began to decrease 3 months post-surgery and was almost completely absent by 6 months post-surgery (LT1M: 5.63%; LT6M: 0.11%). Megamonas, which is involved in organic nutrient metabolism, was absent from pre-surgery to 3 months post-surgery, but began to appear at 6 months post-surgery, with a trend of increasing abundance (LT6M: 2.85%; GC: 5.12%). From the bar charts at each level, we can observe significant differences between the BLT group and the GC group. After undergoing liver transplantation, the gut microbiota composition was significantly disrupted, with this disruption peaking 1 month post-surgery. This trend is consistent with the analysis of Beta diversity. One month post-surgery, the patients' gut microbiota composition began to recover, and by 6 months post-surgery, the proportions of some microbial groups were nearly equivalent to those in the control group. This suggests that the gut microbiota changes after liver transplantation are substantial and closely associated with patient recovery. Notably, 1 month post-surgery represents a critical time point for recovery. Additionally, we performed LEfSe analysis to compare the statistical differences among all samples and constructed phylogenetic trees (Figures 2F, G). The results revealed that a total of 41 bacterial genera across seven groups played a key role. There were four in the BLT group: k__Bacteria, s__Bacteroides_vulgatus, s__Bacteroides_plebeius, and g__Lachnospira; eight in the LT1W group: s__Bacteroides_fragilis, p__Fusobacteriota, c__Fusobacteriia, o__Fusobacteriales, f__Fusobacteriaceae, g__Fusobacterium, p__Proteobacteria, and c__Gammaproteobacteria; two in the LT2W group: g__Rikenellaceae_RC9_gut_group and F__Erysipelatoclostridiaceae; 11 in the LT1M group: o__Lactobacillales, f__Enterococcaceae, g__Enterococcus, f__Lactobacillaceae, g__Lactobacillus, f__Streptococcaceae, g__Streptococcus, s__Streptococcus_salivarius, g__Megasphaera, g__Veillonella, c__Verrucomicrobiae, o__Verrucomicrobiales, f__Akkermansiaceae, and g__Akkermansia; two in the LT3M group: o__Lactobacillales and c__Verrucomicrobiae; three in the LT6M group: o__Lachnospirales, f__Lachnospiraceae, and g__Roseburia; eight in the GC group: s__Prevotella_copri, g__Alistipes, p__Firmicutes, c__Clostridia, g__Agathobacter, o__Oscillospirales, g__Megamonas, and g__Dialister.

### 16S function prediction

We compared all the detected ASVs with the KEGG database for functional prediction analysis to assess the differences in metabolic pathways between the groups. After performing a *T*-test, we generated a map (Supplementary Figure S1) to further compare the compositional components of functional genomics in the KEGG pathways. Compared to the control group, the BLT group had eight significantly enriched modules, the LT1W group had 11, the LT2W group had 11, the LT1M group had 16, the LT3M group had eight, and the LT6M group had three. From these findings, it can be concluded that the LT1M group exhibited the highest enrichment of functional modules compared to the control group, while the LT6M group showed the lowest enrichment of functional modules.

### Metagenomic sequencing

**Figure 3:**
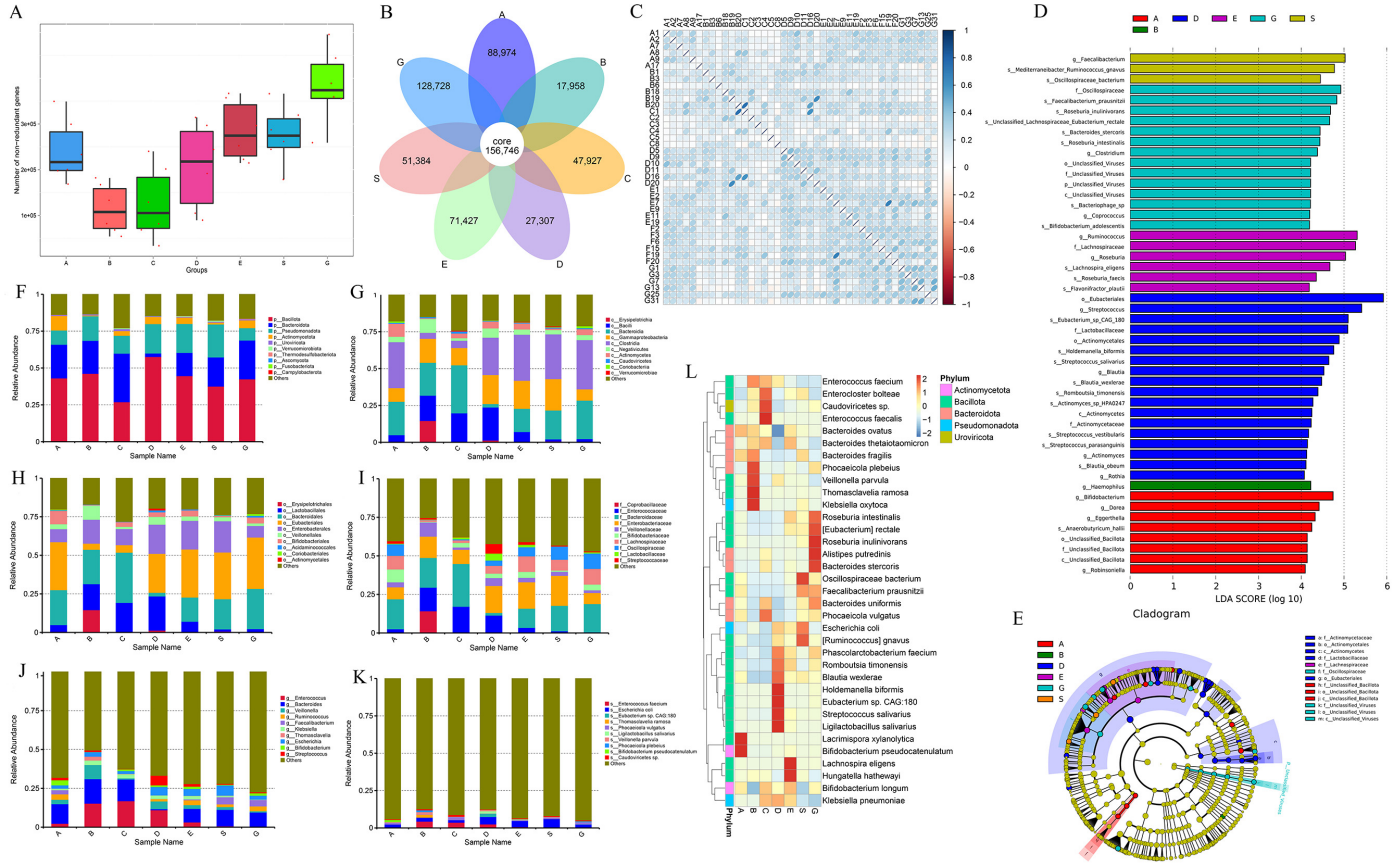
Metagenomic sequencing results and species annotation results. **(A)** Box map of gene number between groups during perioperative period of liver transplantation; **(B)** Venn Diagram; **(C)** Heat map of correlation coefficient between samples; **(D)** LEfSe analysis of bar chart by Metagenomic sequencing; **(E)** LEfse analysis of evolutionary cladistics by Metagenomic sequencing; **(F)** Phylum level species abundance between groups during perioperative period of liver transplantation; **(G)** Class level species abundance between groups during perioperative period of liver transplantation; **(H)** Order level species abundance between groups during perioperative period of liver transplantation; **(I)** Family level species abundance between groups during perioperative period of liver transplantation; **(J)** Genus level species abundance between groups during perioperative period of liver transplantation; **(K)** Species level species abundance between groups during perioperative period of liver transplantation; **(L)** Cluster heatmap of gene number abundance at the species level.

Based on the 16S rRNA sequencing results, we selected six samples from each group using the microPITA Discriminant method, with samples located at the central position (Supplementary Figure S2), totaling 42 samples, for metagenomic sequencing. A total of 1,693,767 gene catalogs (unigenes) were obtained, and a box plot was generated to visualize their distribution (Figure 3A). And we constructed a Venn diagram (Figure 3B) to observe the distribution of gene counts across the seven groups and analyze the shared and unique gene information among different samples (groups),. The total number of shared genes among the seven groups was 156,746. The BLT group (A group) had 88,974 unique genes, the LT1W group (B group) had 17,958 unique genes, the LT2W group (C group) had 47,927 unique genes, the LT1M group (D group) had 27,307 unique genes, the LT3M group (E group) had 71,427 unique genes, the LT6M group (S group) had 51,384 unique genes, and the GC group (G group) had 128,728 unique genes. The results showed that the GC group had the highest gene count among the seven groups, while the LT1W group had the lowest, indicating a significant impact of liver transplantation on the gut microbiota of the patients. Correlation analysis between samples was conducted based on gene count (Figure 3C) to assess the reliability of the experiment and the rationality of sample selection. We observed that, except for the C group, which showed a weaker correlation, all other groups exhibited a strong positive correlation, suggesting that the sample selection was both reasonable and effective.

Consistent with the species analysis method used in the 16S rRNA sequencing results, LEfSe analysis identified 50 species that played a significant role across the seven groups, and their phylogenetic tree was constructed (Figures 3D, E). Compared to the 16S analysis, the metagenomic analysis provided more detailed insights, particularly at the genus and species levels. The GC group exhibited a marked increase in differential bacterial genera, with the addition of viral analysis, such as unclassified_viruses, further enriching the findings. There were eight different microflora in the group before liver transplantation: g__Dorea, g__Eggerthella, s__Anaerobutyricum_hallii, g__Bifidobacterium, g__Robinsoniella, c__Unclassified_Bacillota, o__Unclassified_Bacillota, f__Unclassified_Bacillota. One week after liver transplantation, there was one difference in the bacteria group: g__Haemophilus. There was no difference in microflora 2 weeks after operation. One month after the operation, there were 18 different bacteria genera (the most disrupted time node in the 16S results): s__Eubacterium_sp_CAG_180, s__Blautia_wexlerae, s__Holdemanella_biformis, o__Actinomycetales, f__Lactobacillaceae, s__Streptococcus_vestibularis, g__Blautia, o__Eubacteriales, s__Streptococcus_parasanguinis, s__Romboutsia_timonensis, g__Streptococcus, g__Rothia, f__Actinomycetaceae, c__Actinomycetes, s__Actinomyces_sp_HPA0247, s__Blautia_obeum, g__Actinomyces, and s__Streptococcus_salivarius. There were six different flora 3 months after surgery: s__Lachnospira_eligens, f__Lachnospiraceae, g__Roseburia; g__Ruminococcus, s__Roseburia_faecis, and s__Flavonifractor_plautii. There were three different bacteria groups 6 months after surgery: s__Oscillospiraceae_bacterium, g__Faecalibacterium, s__Mediterraneibacter_Ruminococcus_gnavus. There were 14 different species in GC group: s__Faecalibacterium_prausnitzii, f__Oscillospiraceae, s__Roseburia_inulinivorans, s__Roseburia_intestinalis, s__Bacteroides_stercoris, s__Bacteriophage_sp, s__Bifidobacterium_adolescentis, s__Unclassified_Lachnospiraceae_Eubacterium_rectale, and f__Unclassified_Viruses. Subsequently, we selected the top 10 species at the phylum, class, order, family, genus, and species levels (Phylum, Class, Order, Family, Genus, Species) for each group based on their relative abundance rankings (Table 4) and plotted the relative abundance bar charts of species annotations at different taxonomic levels for each group (Figures 3F–K). Compared to the 16S rRNA results, metagenomic sequencing provides a more detailed analysis at the species level. Upon analyzing the species-level data, we observed that from 1 week to 1 month post-surgery, Enterococcus faecium had a higher proportion (4.37% vs. 2.51%), and Ligilactobacillus and Eubacterium sp. CAG:180 showed significant increases at 1 month post-surgery compared to the other groups (0.02% vs. 1.55% and 0.00% vs. 2.12%, respectively). Building on this, we generated a clustering heatmap of abundance (Figure 3L) to compare the top 35 species at the species level from the 1-month post-surgery group with the other groups. It is evident that, in addition to showing a significant positive correlation with the species we previously analyzed, the 1-month post-surgery group also exhibited a significant negative correlation with species under the phylum Bacteroidota.

**Table 4:** Through metagenomic sequencing sequencing, the species abundance of patients in perioperative liver transplantation groups was displayed at the level of kingdom, phylum, class, order, family, genus and species.

**Taxonomy**	**A**	**B**	**C**	**D**	**E**	**S**	**G**
**Kingdom**							
k__Bacteria	92.88%	94.26%	91.83%	94.21%	93.23%	92.29%	91.54%
k__Viruses	0.93%	0.94%	1.48%	0.58%	0.81%	0.85%	1.27%
k__Eukaryota	0.02%	0.03%	0.04%	0.19%	0.03%	0.04%	0.01%
k__Archaea	0.00%	0.00%	0.00%	0.00%	0.00%	0.00%	0.00%
Others	6.17%	4.78%	6.65%	5.02%	5.93%	6.82%	7.18%
**Phylum**							
p__Bacillota	43.09%	46.20%	26.92%	57.60%	44.60%	37.55%	42.44%
p__Bacteroidota	22.85%	22.42%	32.98%	2.37%	15.84%	19.78%	26.33%
p__Pseudomonadota	9.63%	16.57%	12.11%	19.92%	19.56%	22.34%	8.38%
p__Actinomycetota	9.87%	0.52%	3.20%	4.99%	4.32%	1.27%	5.32%
p__Uroviricota	0.64%	0.82%	1.34%	0.35%	0.49%	0.62%	0.66%
p__Verrucomicrobiota	0.01%	0.01%	0.54%	0.01%	0.43%	0.61%	0.01%
p__Thermodesulfobacteriota	0.08%	0.00%	0.00%	0.01%	0.19%	0.10%	0.40%
p__Ascomycota	0.00%	0.01%	0.01%	0.17%	0.01%	0.01%	0.00%
p__Fusobacteriota	0.00%	0.05%	0.14%	0.01%	0.00%	0.11%	0.00%
p__Campylobacterota	0.01%	0.02%	0.07%	0.01%	0.01%	0.01%	0.01%
Others	13.82%	13.37%	22.69%	14.56%	14.55%	17.60%	16.45%
**Class**							
c__Erysipelotrichia	0.58%	14.59%	0.38%	1.35%	0.51%	0.67%	0.34%
c__Bacilli	4.45%	17.18%	19.43%	22.42%	6.67%	1.44%	1.99%
c__Bacteroidia	22.61%	22.30%	32.56%	2.32%	15.69%	19.60%	26.08%
c__Gammaproteobacteria	9.27%	16.32%	11.77%	19.69%	19.02%	21.31%	7.66%
c__Clostridia	31.16%	3.98%	4.88%	25.33%	31.23%	30.35%	33.36%
c__Negativicutes	3.69%	9.41%	1.75%	6.28%	3.55%	2.93%	3.48%
c__Actinomycetes	8.80%	0.48%	3.16%	4.66%	4.06%	1.00%	3.96%
c__Caudoviricetes	0.64%	0.82%	1.34%	0.35%	0.49%	0.62%	0.66%
c__Coriobacteriia	1.02%	0.04%	0.03%	0.30%	0.24%	0.26%	1.34%
c__Verrucomicrobiae	0.01%	0.01%	0.54%	0.01%	0.43%	0.61%	0.01%
Others	17.78%	14.87%	24.18%	17.28%	18.11%	21.21%	21.10%
**Order**							
o__Erysipelotrichales	0.58%	14.59%	0.38%	1.35%	0.51%	0.67%	0.34%
o__Lactobacillales	4.38%	16.85%	18.97%	22.15%	6.58%	1.42%	1.97%
o__Bacteroidales	22.58%	22.29%	32.51%	2.32%	15.67%	19.59%	26.06%
o__Eubacteriales	31.13%	3.97%	4.88%	25.32%	31.19%	30.30%	33.34%
o__Enterobacterales	8.40%	15.52%	10.43%	18.88%	18.52%	20.28%	7.42%
o__Veillonellales	3.24%	9.15%	1.49%	5.08%	2.86%	2.57%	1.73%
o__Bifidobacteriales	8.64%	0.35%	2.98%	2.93%	3.91%	0.88%	3.79%
o__Acidaminococcales	0.44%	0.26%	0.23%	1.18%	0.68%	0.34%	1.10%
o__Coriobacteriales	0.48%	0.00%	0.02%	0.05%	0.05%	0.20%	1.21%
o__Actinomycetales	0.07%	0.02%	0.00%	1.16%	0.09%	0.04%	0.07%
Others	20.06%	16.99%	28.10%	19.56%	19.93%	23.73%	22.98%
**Family**							
f__Coprobacillaceae	0.21%	14.22%	0.27%	0.36%	0.28%	0.57%	0.08%
f__Enterococcaceae	2.35%	15.33%	16.89%	11.07%	3.18%	0.68%	0.50%
f__Bacteroidaceae	19.43%	19.22%	27.70%	1.65%	12.43%	16.46%	18.29%
f__Enterobacteriaceae	7.76%	13.88%	9.21%	17.57%	17.15%	19.49%	7.17%
f__Veillonellaceae	3.24%	9.15%	1.49%	5.08%	2.86%	2.57%	1.73%
f__Bifidobacteriaceae	8.36%	0.35%	2.98%	2.91%	3.87%	0.86%	3.76%
f__Lachnospiraceae	8.81%	1.11%	1.64%	5.03%	10.07%	7.00%	10.17%
f__Oscillospiraceae	7.49%	0.15%	0.68%	3.44%	5.91%	8.52%	9.95%
f__Lactobacillaceae	0.36%	0.26%	1.30%	4.50%	1.58%	0.03%	0.78%
f__Streptococcaceae	1.60%	1.00%	0.20%	6.21%	1.74%	0.69%	0.66%
Others	40.38%	25.33%	37.63%	42.17%	40.95%	43.13%	46.91%
**Genus**							
g__Enterococcus	2.34%	15.28%	16.84%	11.03%	3.17%	0.67%	0.50%
g__Bacteroides	12.61%	15.77%	13.97%	0.68%	8.77%	10.63%	9.07%
g__Veillonella	2.83%	9.05%	0.74%	5.01%	2.36%	1.77%	0.81%
g__Ruminococcus	3.49%	0.02%	0.02%	1.49%	3.16%	1.73%	3.12%
g__Faecalibacterium	2.65%	0.03%	0.02%	0.05%	0.58%	4.61%	4.13%
g__Klebsiella	0.96%	2.88%	2.59%	2.23%	2.16%	0.90%	0.24%
g__Thomasclavelia	0.07%	2.50%	0.24%	0.08%	0.10%	0.13%	0.01%
g__Escherichia	2.12%	2.87%	1.84%	5.42%	4.45%	6.58%	2.52%
g__Bifidobacterium	3.26%	0.12%	1.06%	1.07%	1.46%	0.34%	1.55%
g__Streptococcus	1.60%	1.00%	0.20%	6.20%	1.73%	0.69%	0.65%
Others	68.07%	50.49%	62.48%	66.74%	72.06%	71.95%	77.39%
**Species**							
s__Enterococcus faecium	0.50%	4.37%	3.67%	2.51%	0.71%	0.04%	0.11%
s__Escherichia coli	1.98%	2.66%	1.71%	4.96%	4.16%	6.08%	2.30%
s__Eubacterium sp. CAG:180	0.00%	0.00%	0.00%	2.12%	0.00%	0.00%	0.16%
s__Thomasclavelia ramosa	0.01%	1.91%	0.00%	0.05%	0.03%	0.08%	0.00%
s__Phocaeicola vulgatus	1.13%	0.19%	2.02%	0.16%	0.37%	0.93%	1.27%
s__Ligilactobacillus salivarius	0.01%	0.02%	0.01%	1.55%	0.19%	0.00%	0.04%
s__Veillonella parvula	0.42%	1.61%	0.14%	0.77%	0.35%	0.34%	0.05%
s__Phocaeicola plebeius	0.01%	1.21%	0.06%	0.00%	0.13%	0.00%	0.61%
s__Bifidobacterium pseudocatenulatum	1.50%	0.00%	0.01%	0.09%	0.24%	0.08%	0.09%
s__Caudoviricetes sp.	0.44%	0.71%	1.20%	0.31%	0.44%	0.50%	0.60%
Others	94.00%	87.33%	91.18%	87.48%	93.40%	91.93%	94.78%

**Figure 4:**
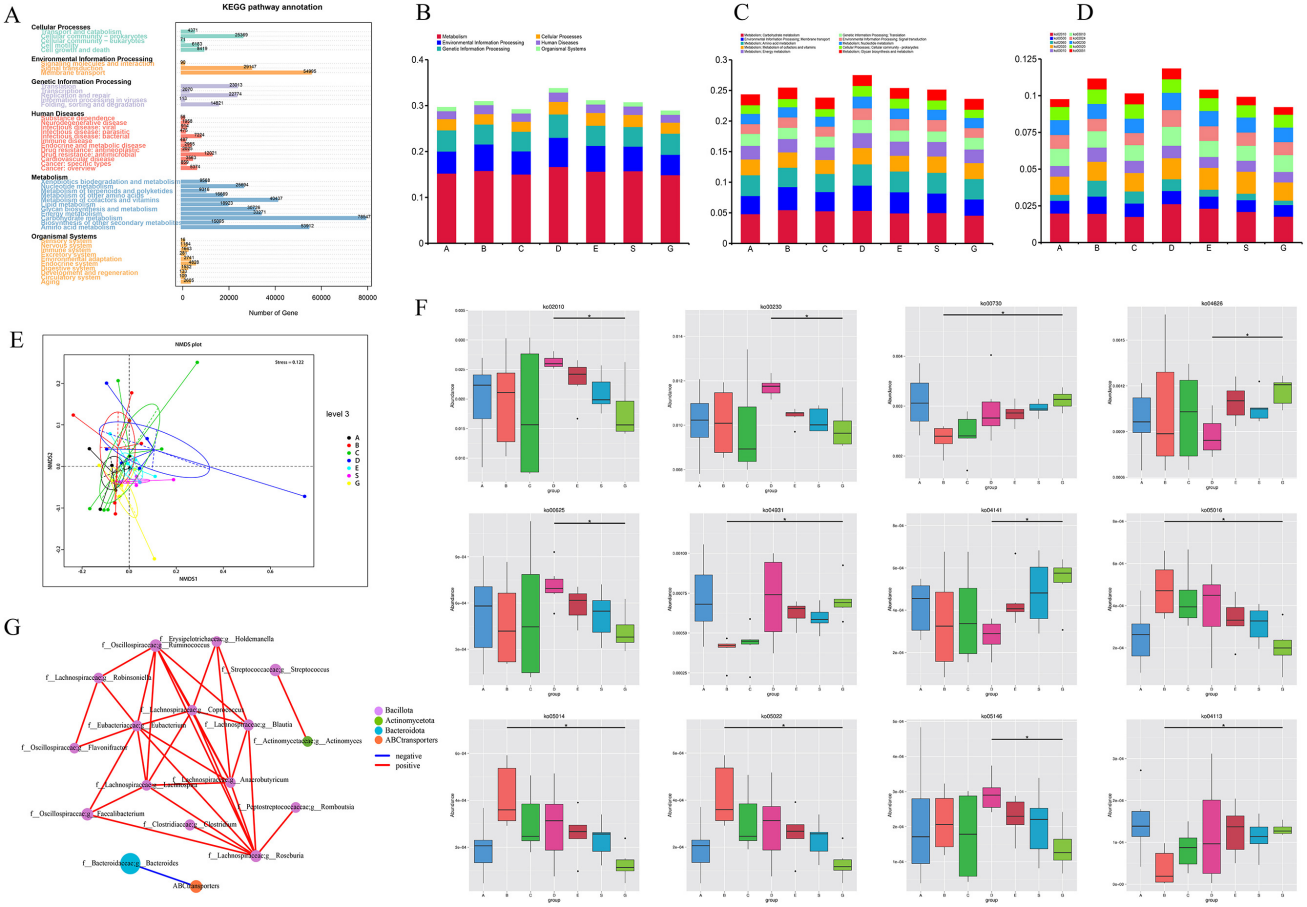
KEGG function results after metagenomic sequencing. **(A)** KEGG LEVEL 1 function distribution of all samples; **(B)** Distribution of KEGG LEVEL 1 in each group during perioperative period of liver transplantation; **(C)** Distribution of KEGG LEVEL 2 in each group during perioperative period of liver transplantation; **(D)** Distribution of KEGG LEVEL 3 in each group during perioperative period of liver transplantation; **(E)** NMDS analysis based on functional abundance; **(F)** Metastat analysis of functional differences among KEGG LEVEL 3 groups (top 12); **(G)** Correlation network analysis of ko02010 specific functional pathways and related genera (* means *p* < 0.05).

Additionally, we performed functional annotation of the genes using the KEGG database and generated statistical plots (Figure 4A). The pathway with the highest functional abundance at LEVEL One was Metabolism, with the highest abundances observed in carbohydrate metabolism and amino acid metabolism. The next most abundant pathway was Environmental Information Processing, with the membrane transport pathway showing the highest abundance. The relative abundance of the top 10 pathways at LEVEL One, LEVEL Two, and LEVEL Three for each group is shown in the figures (Figures 4B–D). It can be observed that at all levels, the functional relative abundance is highest at 1 month post-surgery, indicating that the gut microbiome is more active and complex during this period. In LEVEL Three, the pathway with the highest functional abundance is the Ko02010 pathway, suggesting that this pathway plays a major role in the gut microbiome function of liver transplant recipients. The functional distribution of each group at LEVEL Three (Figure 4E) by NMDS dimensionality reduction analysis can further support our hypothesis that there are certain differences between the control group and the pre-liver transplant group. Post-surgery, the functionality of the gut microbiota in liver transplant recipients showed significant changes, which corresponded with changes in the gut microbiota composition. These functional differences were most pronounced 1 month post-surgery, with the greatest degree of functional variability observed at that time. Following this, the gut microbiota functionality gradually aligned with that of the control group as the post-surgery period extended. However, 6 months post-surgery did not mark the endpoint of gut functionality recovery in liver transplant patients. In addition, to investigate the functions with significant differences between groups, we performed hypothesis testing on the functional abundance data between groups using the Metastats method based on the relative abundance table at the LEVEL Three taxonomy. This allowed us to obtain *p*-values. Afterward, we corrected the *p*-values to obtain q-values. Finally, we selected the functions with significant differences based on the *q*-values and plotted a boxplot depicting the abundance distributions of the top 12 differential functions between groups (Figure 4F).

The pathway with the highest abundance difference was ko02010, which showed significant differences between the control group and the 1e-month post-surgery group. Additionally, based on previous studies of the abundance statistics for LEVEL Three pathways, ko02010 is also among the top 10 most abundant pathways at LEVEL Three. Therefore, we believe that this pathway may be an important one influencing the changes and recovery of gut microbiota function during the perioperative and postoperative periods in liver transplantation. The main protein encoded by this pathway is an ABC transporter.

To further investigate the relationship between the gut microbiota and this pathway, we performed a Spearman correlation network analysis (Figure 4G) between the ko02010 pathway and 22 specific genera from the 50 differential genera identified through LEfSe analysis at the genus level. The results were intriguing: this pathway showed a significant negative correlation only with g__Bacteroides under the phylum Bacteroidota, while no significant correlation was found with genera under the phyla Bacillota and Actinobacterota.

We observed this pattern in the abundance table of various microbiota. Specifically, from 2 weeks to 1 month post-surgery, o__Bacteroidota (31.51% vs. 2.32%), f__Bacteroidaceae (27.70% vs. 1.65%), and g__Bacteroides (13.97% vs. 0.68%) showed a marked decrease in proportion, while their proportions significantly increased again at 3 months post-surgery (Figures 3H, I, G). Similarly, in the 16S rRNA amplicon sequencing analysis, we also observed the same result. Therefore, we hypothesize that the abnormal changes in g__Bacteroides 1 month post-surgery may be a key factor leading to the functional abnormalities of the ko02010 pathway during this period. Additionally, the abnormal changes in g__Bacteroides during this time may also contribute to the gut microbiota dysbiosis observed in liver transplant recipients. Hence, we consider g__Bacteroides to be an important microbiota group influencing postoperative recovery in liver transplant patients.

### Correlation analysis between microflora and clinical indicators

**Figure 5:**
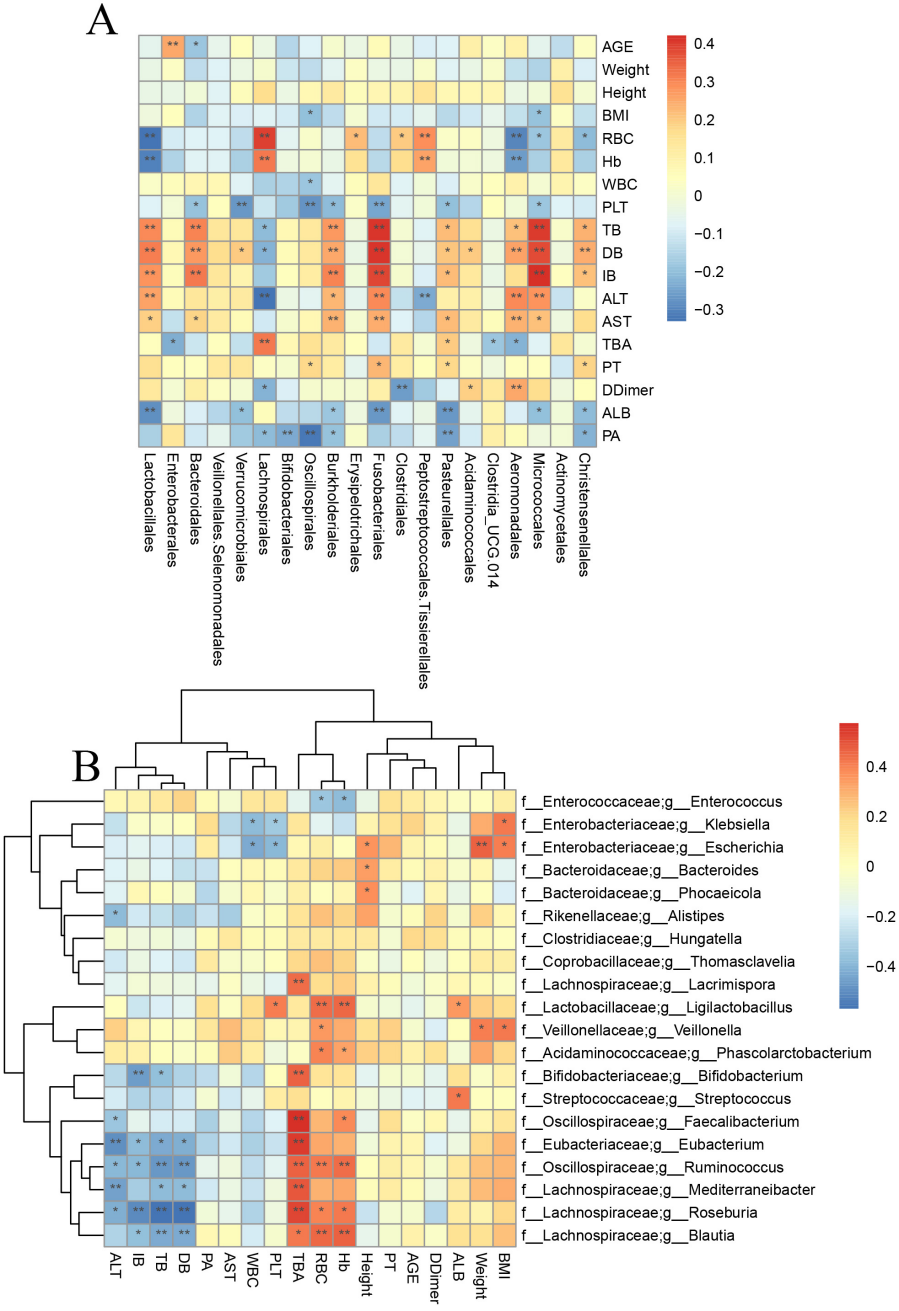
Correlation analysis between specific bacteria and clinical indicators. **(A)** Correlation analysis between 16s rRNA sequencing results and clinical indicators. **(B)** Correlation analysis between metagenomic sequencing results and clinical indicators (* means *p* < 0.05; ** means *p* < 0.01).

To explore the relationship between the gut microbiota and clinical indicators in liver transplant patients during the perioperative period, we conducted a Spearman correlation analysis between the clinical indicators of all samples and the top 20 genera at the genus level (Figure 5A). By analyzing the correlation between the 16S rRNA sequencing results and clinical indicators, we found that bilirubin metabolism was closely associated with the gut microbiota. For example, Lactobacillus, Bacteroides, Fusobacteriales, and Micrococcaceae showed a significant positive correlation (*p* < 0.05).

In addition, Enterococcus showed a significant negative correlation with HB, RBC, and ALB (*p* < 0.01). To obtain more precise and representative results, we performed a correlation analysis and clustering analysis between the metagenomic sequencing results and clinical indicators (Figure 5B). The results showed that Bifidobacterium, Lachnospira, Roseburia, Blautia, and Eubacterium were significantly negatively correlated with bilirubin metabolism (*p* < 0.05) and significantly positively correlated with bile acid metabolism (*p* < 0.05). However, Bacteroides did not show any significant correlation with clinical indicators in the metagenomic sequencing results.

## Discussion

In this study, we conducted 16S rRNA sequencing on 151 gut microbiota samples and found that the gut microbiota of liver transplant patients underwent dysbiosis. The most severe dysbiosis occurred 1 month post-surgery, after which the gut microbiota gradually started to recover toward the healthy control group. At this time, the vast majority of patients have been discharged from the hospital, resumed their normal diet, and stopped using antibiotics. These clinical changes may be closely related to our results. By 6 months post-surgery, the microbiota was closest to that of the healthy control group, but this was not the endpoint of the patient's gut microbiota recovery. Our team collected follow-up data from 123 LT patients at 6 months post-surgery, with mortality rates of 3.25%, cytomegalovirus hemophilia patients 0.81%, and diabetes patients 25.93%.

Based on this, we selected 42 gut microbiota samples for metagenomic sequencing using the microPITA method, and the results were similar to those obtained from the 16S rRNA sequencing.

Further analysis using LEfSe identified 50 bacterial genera that play a significant role during the perioperative and postoperative periods in liver transplant patients. Most of the bacterial communities, such as Enterococcus and Lactobacillus, showed an increase first and then a decrease, while Bacteroides showed a decrease first and then an increase. Both reached a peak (or a bottom) 1 month after surgery. Enterococcus is often associated with infection after liver transplantation and is an important bacterial community that affects recovery after liver transplantation (Liu et al., 2024). The abundance of this bacterial community was the highest in patients 1 month after the transplantation. The role of Clostridia in human metabolism is significantly diverse. Some strains, as components of the intestinal flora, participate in physiological processes such as human material metabolism and intestinal homeostasis regulation. For instance, Clostridium butyricum can produce butyric acid, which can promote the proliferation and repair of intestinal mucosal cells. Strengthen the intestinal barrier function. Pathogenic strains, on the other hand, cause diseases by disrupting normal metabolism. Moreover, studies have found that patients after liver transplantation have a high risk of Clostridium difficile infection, which can easily lead to hyperbilirubinemia and transplantation failure after the operation. Lactobacillus is an important bacterial community that protects the liver from damage caused by cholestasis by increasing bile acid excretion (Wu et al., 2024). Typically, patients have gradually resumed a normal diet by 1 month postoperatively. However, according to our study, this period still carries a higher risk of infection and related complications.

A healthy gut microbiota is primarily composed of Firmicutes and Bacteroidetes. The ratio between these two phyla (F/B ratio) is commonly used to reflect susceptibility to diseases (Wong et al., 2022). After liver transplantation, the F/B ratio gradually increases, reaching its peak at 1 month post-transplant, and then gradually decreases. This suggests that patients are at a higher risk of infection during the first month following liver transplantation. In addition, Firmicutes have important biological functions, such as synthesizing short-chain fatty acids (SCFA), which are essential for liver regeneration. Some scholars have shown (Ley et al., 2006) that a decrease in Firmicutes and an increase in Proteobacteria will lead to a decrease in SCFA, thereby inhibiting liver regeneration. Our study found that within 1 month after liver transplantation, there were fewer Firmicutes and more Proteobacteria. During this period, liver regeneration function would be inhibited due to the reduction of SCFA production and the rational use of immune rejection drugs would help the liver better adapt to the new host. In addition, the stability of bile salt metabolism can not only maintain the stable metabolic state of intestinal microecology, but also reduce liver damage. The function of Faecalibacterium has been proven to be closely related to fecal bile acid metabolism. Faecalibacterium can decompose butyrate, activate the NF-kb signaling pathway, and induce the production of inflammatory mediators related to Treg-cell stimulation. Therefore, Faecalibacterium is closely related to the stability of the immune status of liver transplant patients (Yin et al., 2023; Carr et al., 2023). In our study, through metagenomic sequencing, we observed that Faecalibacterium is suppressed to a relatively low proportion within the first month post-transplantation, and it only begins to increase after 3 months. Other gut microbiota involved in bile acid metabolism, such as Akkermansia, which are important probiotics in the human body, exhibit a similar trend of change (Lee et al., 2022). Additionally, some antibiotic-resistant bacteria (Bárcena et al., 2019), such as Enterococcus, which is a key member of the ESKAPE pathogens, also showed an increase at 1 month post-surgery, followed by a decrease after 1 month. Therefore, changes in the microbiota during this period may contribute to hospital-acquired infections. One month after liver transplantation (LT) is the period when the gut microbiota is most disordered. During this time, appropriate treatment can promote the restoration of the gut microbiota, facilitate the adaptation of the new liver, and accelerate the postoperative recovery of liver transplant patients.

In recent years, the impact of gut microbiota on liver diseases has become a hot research topic, with the gut microbiota playing an important role in the onset and progression of diseases. Our study is the first to identify that 1 month post-liver transplantation is the time point when liver transplant patients experience the most severe gut microbiota dysbiosis. After this, the gut microbiota gradually recovers toward that of the healthy control group. As early as 2017, Sun et al. (2017) found that the fecal microbiota community was significantly altered due to the impact of liver transplantation. Our team also discovered in 2022 that (Lai et al., 2022), 1 and 2 weeks post-liver transplantation, the gut microbiota of patients underwent severe dysbiosis at both time points. However, these results only have 16S sequencing and lack metagenomics, so further research on its function and pathways is needed. At the same time, some Japanese scholars have found (Kato et al., 2017) that by collecting fecal samples from 38 perioperative patients, the diversity of the intestinal flora began to decline within 3 weeks after the operation. This is consistent with our discovery that 1 month after the operation is the turning point for the recovery of intestinal flora in liver transplant patients. Meanwhile, the number of our patients has increased to 59 compared to before, and the follow-up period has also been extended to more than 6 months. We initially explored the recovery status and trend of the intestinal flora in liver transplant patients, and identified the key time points for their recovery. From the results, we can see that liver transplantation can indeed benefit patients. The period of 1 month is the most disordered for the intestinal flora of liver transplant patients, and by 6 months after the operation, the intestinal flora of the patients has gradually recovered to that of the control group. The findings of this study provide ideas for the rapid recovery and reduction of complications of clinical liver transplant patients.

In addition, Lai et al. (2022) confirmed that the composition of the gut microbiota post-liver transplantation is closely related to the severity of the disease and the use of antibiotics. Furthermore, the extensive use of immunosuppressants also significantly affects changes in the gut microbiota (Vega-Abellaneda et al., 2024). Dysbiosis of the gut microbiota post-liver transplantation can lead to various complications, such as post-transplant metabolic diseases, post-transplant hepatic encephalopathy, and post-transplant hepatocellular carcinoma (Zhang et al., 2018). Additionally, some researchers have suggested that analyzing the gut microbiota can help predict the prognosis of liver transplant patients (Davin-Regli et al., 2019). Therefore, exploring the changes in the gut microbiota after liver transplantation is of great importance for the prompt recovery of liver transplant patients. However, current research on the changes in gut microbiota post-liver transplantation has a limited sample size and lacks long-term studies.

We also performed gene function annotation and identified 10 pathways with higher abundance. By using the Metastats method, we identified the top 12 pathways with significant differences. The analysis concluded that ABC transporters may be an important pathway affecting the recovery of LT patients. It was found that this pathway showed a significant negative correlation with Bacteroides.

Gut microbiota dysbiosis can also lead to dysfunction. ABC transporters are membrane proteins that play important roles both in the gut microbiota and in liver metabolism. ABC transporters in gut bacteria help to transport exogenous and endogenous substances, reducing the burden of harmful substances on the body while maintaining the abundance and diversity of the gut microbiota (Dietrich et al., 2003). Among ABC transporters, ATP-binding cassette subfamily B member 4 (ABCB4) is involved in the efflux of bile salts in gut bacteria and also influences the intestinal absorption of vitamin D, thereby preventing liver fibrosis (Hochrath et al., 2014). ABC transporters in intestinal bacteria strongly affect drug absorption and pharmacokinetics. They contribute to increased resistance to antimicrobial agents, including antibiotics and host-derived antimicrobial peptides (Villanueva et al., 2019). These transporters actively pump antimicrobial compounds out of bacterial cells, reduce their intracellular concentrations, and protect bacteria from the harmful effects of these compounds, allowing bacteria to survive in the presence of various antimicrobial agents encountered in the intestinal environment. In addition, some ABC transporters promote the transport of short-chain fatty acids (SCFAs) produced by intestinal bacteria (Lan et al., 2023), which play an important role in host liver physiology and immune regulation. In our study, we found that the abundance of ABC transporters in the intestinal flora was significantly increased compared with the control group and other groups at 1 month after LT. If the expression level of ABC transporters is too high, lipid metabolism will be dysregulated. For example, ABCA1 and ABCG1 play a vital role in maintaining lipid homeostasis. Overexpression of these transporters can disrupt lipid metabolism, leading to abnormal accumulation or depletion of lipids in cells and tissues, thereby causing complications of cardiovascular and endocrine diseases after liver transplantation (Moustafa et al., 2004). ABC transporters such as ABCB4 (MDR3) are also responsible for transporting phospholipids into bile, which is essential for the formation of bile and the maintenance of bile flow (Morita et al., 2019). Overexpression of ABC transporters involved in bile secretion may lead to excessive phospholipid secretion, altering the composition of bile and potentially causing biliary complications after liver transplantation. We speculated that the changes in ABC transporter function 1 month after liver transplantation may affect the stability of the gut microbiota during this period, which in turn influences the postoperative recovery of liver transplant patients. This could be a key factor affecting their postoperative recovery and long-term outcomes.

Bacteroides is a genus of Gram-negative anaerobic bacteria commonly found in the human gastrointestinal tract. They play a critical role in the digestion and breakdown of complex carbohydrates and are involved in the production of various metabolites, including short-chain fatty acids (SCFAs) such as acetate, propionate, and butyrate. These SCFAs are essential for maintaining gut health, as they provide energy to colonocytes, support the integrity of the intestinal barrier, and regulate immune responses. Additionally, Bacteroides are involved in the modulation of gut microbiota composition, and their abundance can influence the overall microbial ecosystem and host health. These metabolites can be absorbed into the bloodstream and reach the liver, where they play a crucial role in influencing liver metabolism and function. Studies have shown that Bacteroides disorders are often associated with adverse outcomes after liver transplantation (Wong et al., 2022). In recent years, many studies have found that the metabolism of Bacteroides plays an important role in regulating intestinal flora and liver metabolism. Zhang's study found that in the process of inducing NAFLD in mice by high fat and high cholesterol, the intestinal flora was significantly disturbed, and Bacteroides was significantly depleted (Zhang et al., 2021). Other studies have shown that Bacteroides is significantly correlated with intrahepatic triglycerides and liver enzymes (Ni et al., 2023). Immune responses can also contribute to gut microbiota dysbiosis after liver transplantation. Jiang et al. found that the use of immunosuppressants (tacrolimus) after liver transplantation leads to a reduction in Bacteroides, thereby affecting the stability of gut function (Jiang et al., 2018).

Other scholars have found that in acute rejection after orthotopic liver transplantation, the intestinal flora is dominated by excessive growth of Bacteroides and Ruminococcus. Therefore, studies have shown that Bacteroides plays a significant role after liver transplantation (LT). Our research found that its abundance initially decreases and then increases post-LT. Specifically, at 1 month post-transplantation, Bacteroides reaches its lowest point. Changes in Bacteroides during this period will affect liver metabolism and immune responses, subsequently influencing the treatment outcomes and complications in liver transplant (LT) patients. Therefore, Bacteroides plays a crucial role in liver transplantation.

The study concluded with Spearman correlation analysis, which revealed that ABC transporters were significantly negatively correlated only with Bacteroides. No significant correlation was found with other microbial populations that differed during the perioperative and postoperative periods in liver transplant patients. There are few reports on the correlation between the two. Dai et al. found through intestinal flora and metabolic profiles that the increase in F/B affects arachidonic acid metabolism and linoleic acid metabolism, leading to the accumulation of unsaturated fatty acids in the intestine. In addition, the decrease in this ratio also leads to the inhibition of the PPAR signaling pathway and the stimulation of ABC transporters, which affects lipid synthesis and fat decomposition, leading to adverse reactions such as liver damage, ileal inflammation, and weight loss in mice (Dai et al., 2022). The increase in Bacteroides has a reducing effect on the expression of ABCA1, and its expression is mainly regulated by liver X receptors (Badi et al., 2020). Finally, through correlation analysis with clinical indicators, the 16S rRNA results showed a significant positive correlation between Bacteroides and bilirubin metabolism. This finding further supports our hypothesis regarding the connection between Bacteroides and ABC transporters. However, in the metagenomic sequencing analysis, Bacteroides did not show a strong correlation. Further studies with larger sample sizes and optimized experimental methods are needed to explore the mechanism of interaction between Bacteroides and ABC transporters, providing additional insights for the clinical diagnosis and treatment of liver transplant (LT) patients. Our study is the first to establish a correlation between Bacteroides and ABC transporters in liver transplant (LT) patients. We hypothesize that due to the surgical impact and the extensive use of immunosuppressive drugs after liver transplantation (LT), the abundance of Bacteroides decreases, leading to a reduction in the production of gut-derived SCFAs and secondary bile acids. This disruption affects liver lipid and bile acid metabolism, activates LXRs, and subsequently increases the expression of intestinal ABC transporters, thereby promoting the transport and metabolism of lipids and bile acids within the gut.

However, there are still several limitations in this study. First, this experiment only explored the differences in the gut microbiota of LT patients at different time points post-surgery, and the correlation between these differences and clinical outcomes has not been thoroughly investigated. Second, the validation method used was a cross-sectional study, and future research should incorporate prospective experiments to further validate the patterns of gut microbiota changes at different postoperative stages in LT patients. In addition, future experiments should further explore the functional mechanisms of gut microbiota and investigate the correlation between microbiota-derived metabolites and recovery in LT patients post-surgery.The sample size is relatively small, and the included cases are predominantly derived from a single medical institution, resulting in limited geographical representation. This limitation may compromise the generalizability of the conclusions. Future studies are recommended to expand the sample size through multi-center collaboration and extend the follow-up period beyond 1 year to support more comprehensive analyses.

## Conclusion

This study collected stool samples from liver transplant (LT) patients during the perioperative and postoperative recovery periods to explore the changes in the gut microbiota of LT patients. Using 16S rRNA sequencing technology, we compared the diversity, abundance, and composition of the gut microbiota at different time points in liver transplant patients. We observed that the gut microbiota of LT patients initially became increasingly disrupted after surgery and then gradually recovered toward the levels observed in the control group. Notably, our study is the first to identify that 1 month post-liver transplantation is the period when the gut microbiota is most disrupted. And through metagenomic sequencing, we validated the conclusions derived from 16S rRNA sequencing and identified a correlation between Bacteroides and ABC transporters. Furthermore, we found that Bacteroides plays a significant role in the recovery process of LT patients. This study is expected to provide important theoretical evidence and treatment strategies for reducing complications and improving the quality of life after liver transplantation, while also offering guidance for our team's future research. We believe that, in the future, research in this field will contribute to better management and treatment of liver transplant patients post-surgery.

## Data Availability

The original contributions presented in the study are publicly available. This data can be found at: https://www.ncbi.nlm.nih.gov/, PRJNA1363135.
